# Supplementary material for: Chromatin compaction during confined cell migration induces and reshapes nuclear condensates
Source: Nat Commun. 2024 Nov 18;15:9964. doi: 10.1038/s41467-024-54120-5 (PMC11574006; doi:10.1038/s41467-024-54120-5)
Supplement: Supplementary file 1 — Supplementary Information [file 41467_2024_54120_MOESM1_ESM.pdf]

Supplementary Information for

Chromatin Compaction During Cancer Cell Confined Migration Induces and Reshapes Nuclear Condensates

Jessica Z. Zhao[1], Jing Xia[1], Clifford P. Brangwynne[1,2,3,4,*]

[1]Department of Chemical and Biological Engineering, Princeton University, Princeton, NJ 08544
[2]Princeton Materials Institute, Princeton University, Princeton, NJ 08544
[3]Howard Hughes Medical Institute, Princeton University, Princeton, NJ 08544
[4]Omenn-Darling Bioengineering Institute, Princeton University, Princeton, NJ 08544

[*]To whom correspondence may be addressed. Email: cbrangwy@princeton.edu

Table of Content

Supplementary Discussion
Supplementary Figures
Supplementary References

Supplementary Discussion

Mechanical Characterization of Chromatin

Besides particle tracking of Corelet droplets, to further examine the mechanical nature of chromatin during confined migration, we also performed experiments using different probes: Nuclear Genetically Encoded Multimeric nanoparticles (nucGEMs), with a 40-nm size whose bright fluorescence allows single particle tracking (**Supplementary Data Fig. S3a**)[1]. We find no difference in the pairwise MSD of single nucGEMs in the leading vs. trailing half; this suggests that the differences detected by monitoring fluctuating droplet motion are dictated by chromatin with apparent mesh sizes larger than 40nm, or potentially only manifest on experimentally longer time scales (**Supplementary Fig. S3b**). However, due to limitation of particle tracking and bleach-prone nature of nucGEMs, we could not measure the MSD at a longer timescale more relevant for condensate formation in the nucleus, which is roughly tens of seconds[2,3]. Therefore, we performed fluorescence recovery after photobleaching (FRAP) experiment in ferritin-core expressing cells. Using a 500-nm ROI in both advancing and trailing half of the same cell (**Supplementary Fig. S4a**), we find that averaged FRAP curves show a higher mobile fraction for the trailing half compared to advancing half (**Supplementary Fig. S4b**). Taken together, the Corelet microrheology data and FRAP results are consistent with the trailing half of the constrained nucleus being a mechanically softer, heterogeneous material.

Supplementary Figures

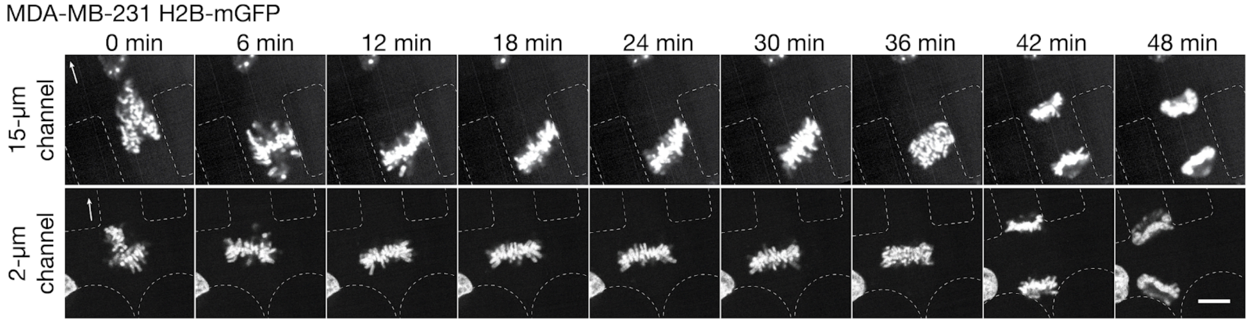

Supplementary Fig. S1. Representative stills of mitosis events of cells after having passed through 15-µm channel (top) and 2-µm channel (bottom). Dashed white borders mark locations of PDMS pillars. White arrows indicate migration direction set by 10% FBS gradient. Scale bar: 5-µm.

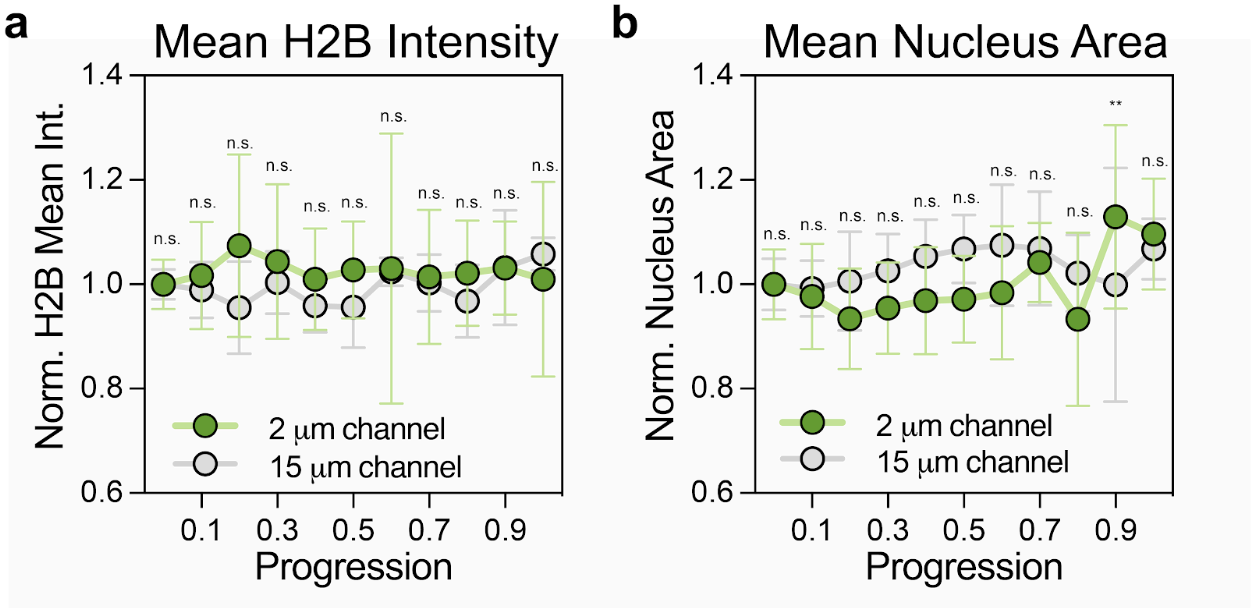

Supplementary Fig. S2. (a) Plot of normalized H2B mean intensity as nuclei progress through constrictions. Error bars are SD of 13 and 24 cells from N = 3 independent experiments, for 15-μm and 2-μm channels respectively. Statistics significance by one-way ANOVA (two-tailed) comparing measurements of 2-μm channel group with 15-μm channel group at each progression bin. (b) Plot of normalized nucleus area as nuclei progress through constrictions. Error bars are SD of 13 and 24 cells from N = 3 independent experiments, for 15-μm and 2-μm channels respectively. Statistics of each progression bin compares measurement for 15-μm group vs 2-μm group. **p = 0.006 by one-way ANOVA test (two-tailed).

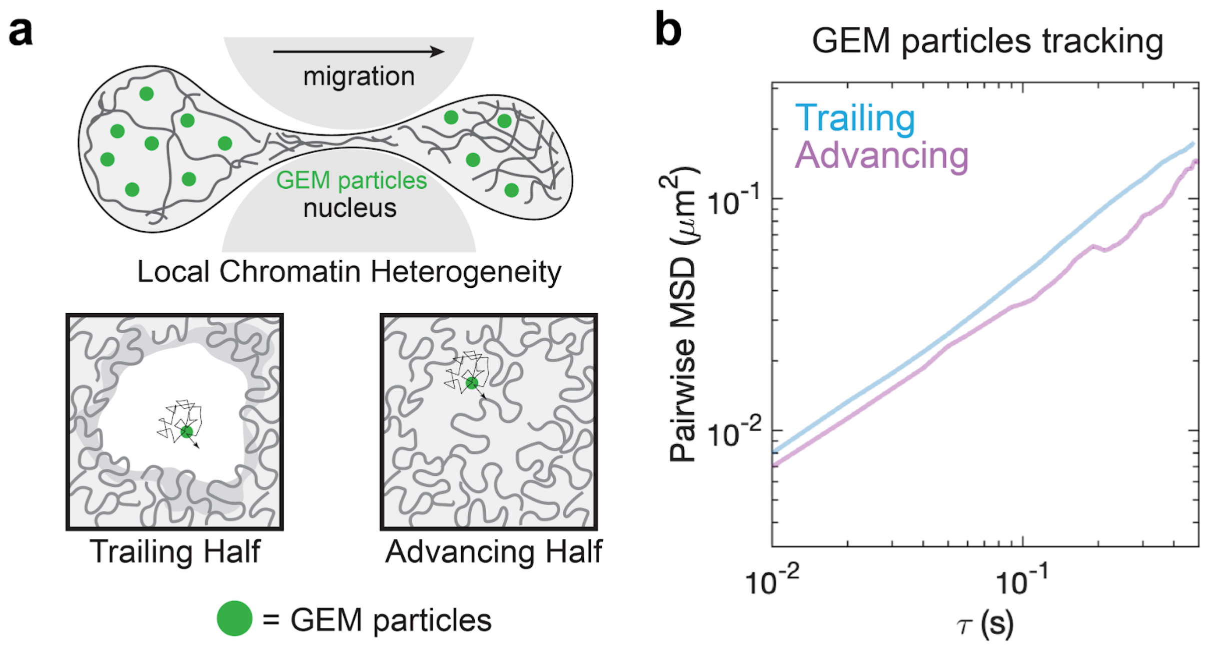

Supplementary Fig. S3. Mechanical characterization of trailing vs advancing half of nucleus. (a) Schematics of varied chromatin heterogeneity contributing to differential mechanics within single cell nucleus, probed by GEM nanoparticles diffusing in cell nucleus. (b) Chromatin mechanics probed by nucGEMs nanoparticles in the advancing (purple) vs trailing half (blue) of the confined nucleus. Population-averaged pairwise MSD (n = 13 cells, N = 3 independent experiments) of particles in the advancing vs. trailing half.

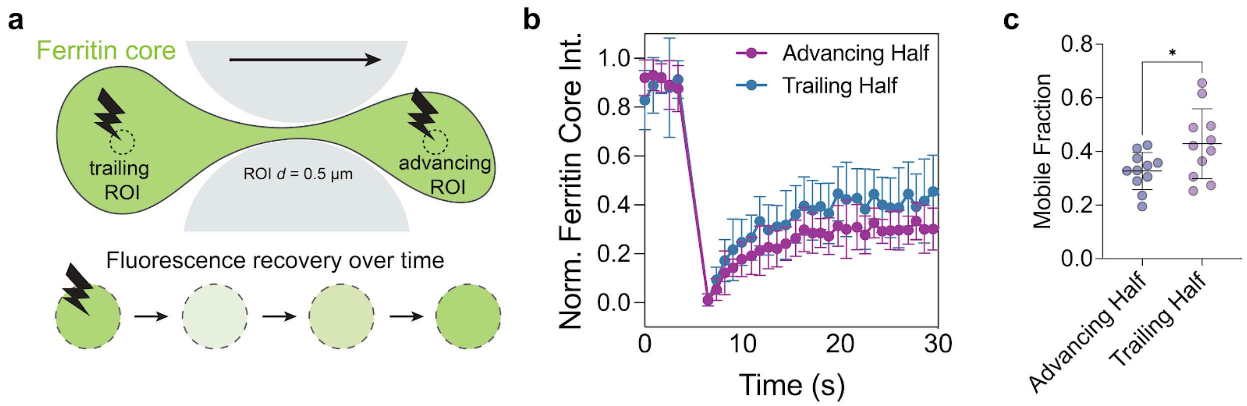

Supplementary Fig. S4. Additional mechanical characterization of trailing vs advancing half of nucleus using fluorescence recovery after photobleaching (FRAP). (a) FRAP of ferritin core of the same cell in a 500-nm ROI in either the advancing or trailing half of the nucleus at Progression = 0.5. (b) Average fluorescence recovery curve normalized to a non-bleached ROI. Error bar shows S.D. of 12 ROIs across 12 cells, N = 3 independent experiments. (c) Mobile fraction quantified by fitting FRAP recovery curve. Statistics done by paired t test (two-tailed). *p = 0.021.

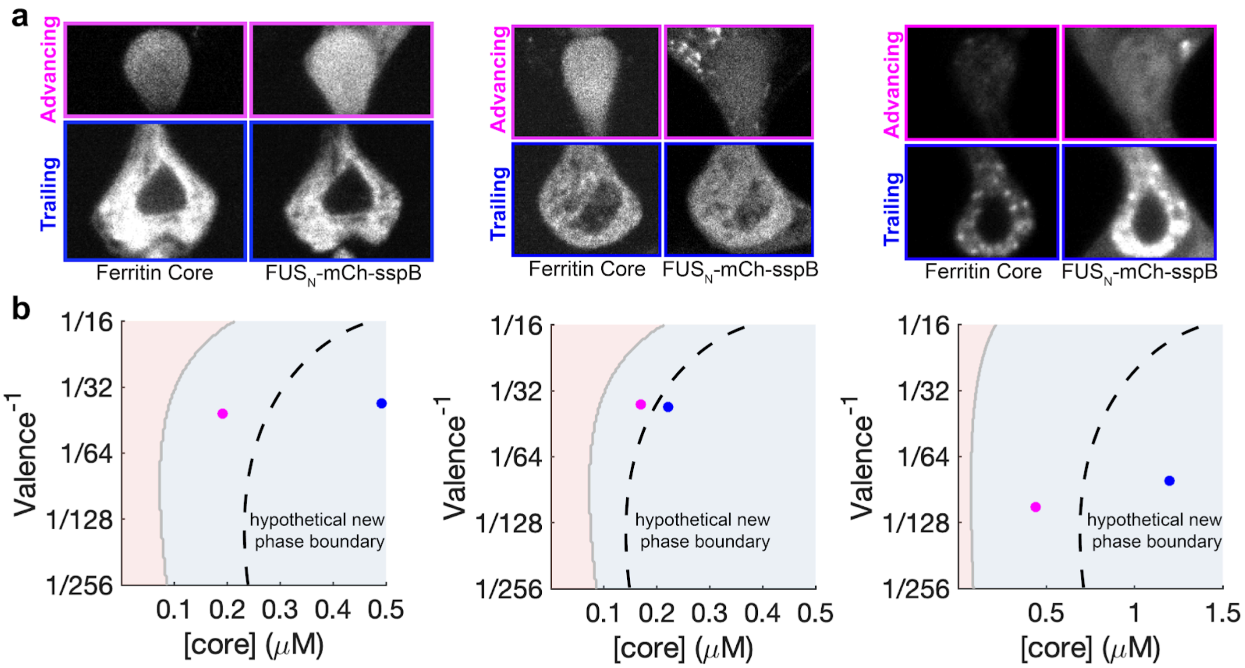

Supplementary Fig. S5. Examples of Corelets component redistribution in the advancing vs. trailing half of cell nucleus. (a) Pre-light activation snapshots of Corelet components, ferritin core and IDR-sspB, in cells in 2-μm constrictions. (b) Phase mapping of individual cells on top of the phase diagram binodal line. Solid gray line: binodal line mapped unconfined cells. Dashed black line: hypothetical binodal line after phase boundary shift upon mechanical deformation with constriction.

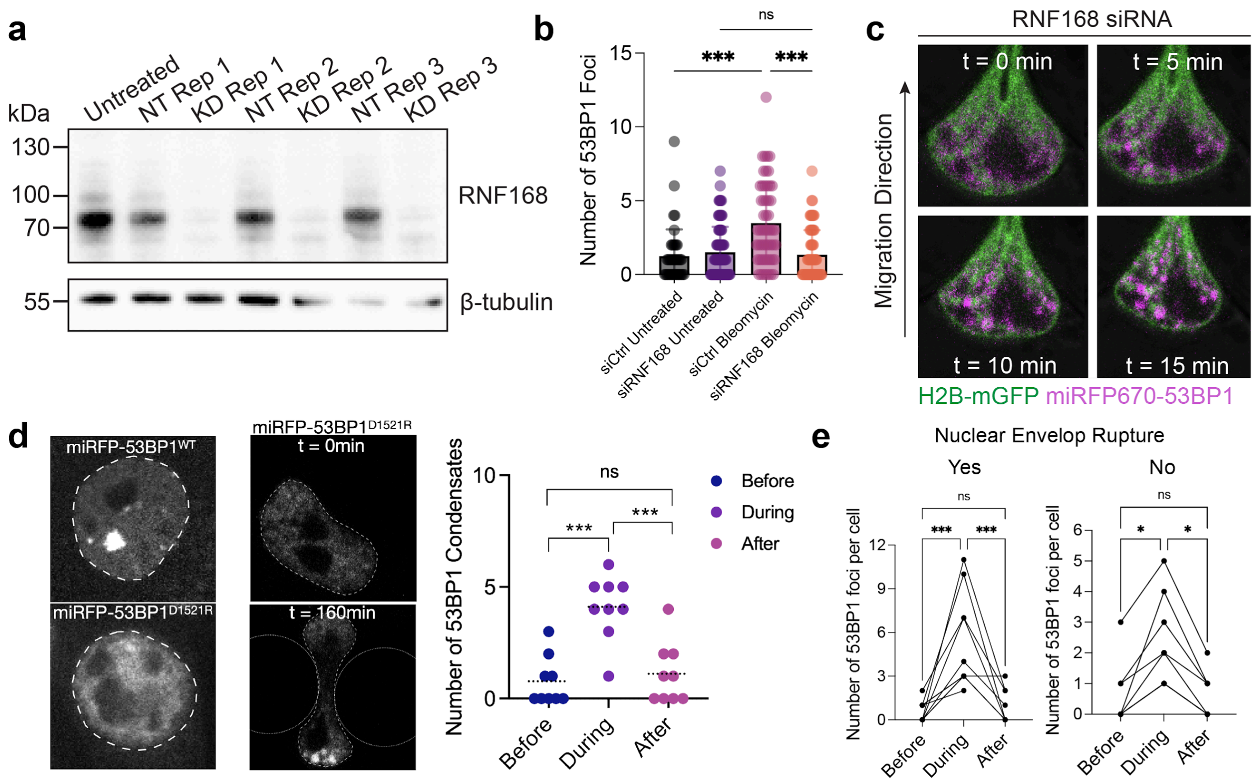

Supplementary Fig. S6. *De novo* 53BP1 condensates can form independent of DNA damage repair function. (a) Western blot validation of knockdown of a DNA double-stranded break (DSB) repair protein RNP168 upstream of 53BP1 via siRNA. N = 3 independent experiments. (b) Number of 53BP1 foci quantified by segmenting images of anti-53BP1 immunofluorescence staining. ***p < 0.001 by one-way ANOVA test (two-tailed). (b) Representative images of siRNF168 treated cells expressing H2B-mGFP (green) and miRFP670-53BP1 (magenta) undergoing confined migration show *de novo* 53BP1 foci formation. (c) Representative images of exogenous expression of full-length 53BP1 protein with a Tudor domain point mutation D1521R shows *de novo* 53BP1 foci formation. (d) Condensation of 53BP1^{D1521R} is reversible. ***p < 0.001 by one-way ANOVA test. n = 9 nuclei from N = 3 independent experiments. (e) Reversible condensation of 53BP1 during confined migration is independent of nuclear envelope (NE) rupture events. n = 17 from N = 3 independent experiments. NE rupture is called by observing miRFP670-53BP1 signal leakage into the cytoplasm during confined migration events. *p = 0.02 and ***p < 0.001 by one-way ANOVA test (two-tailed).

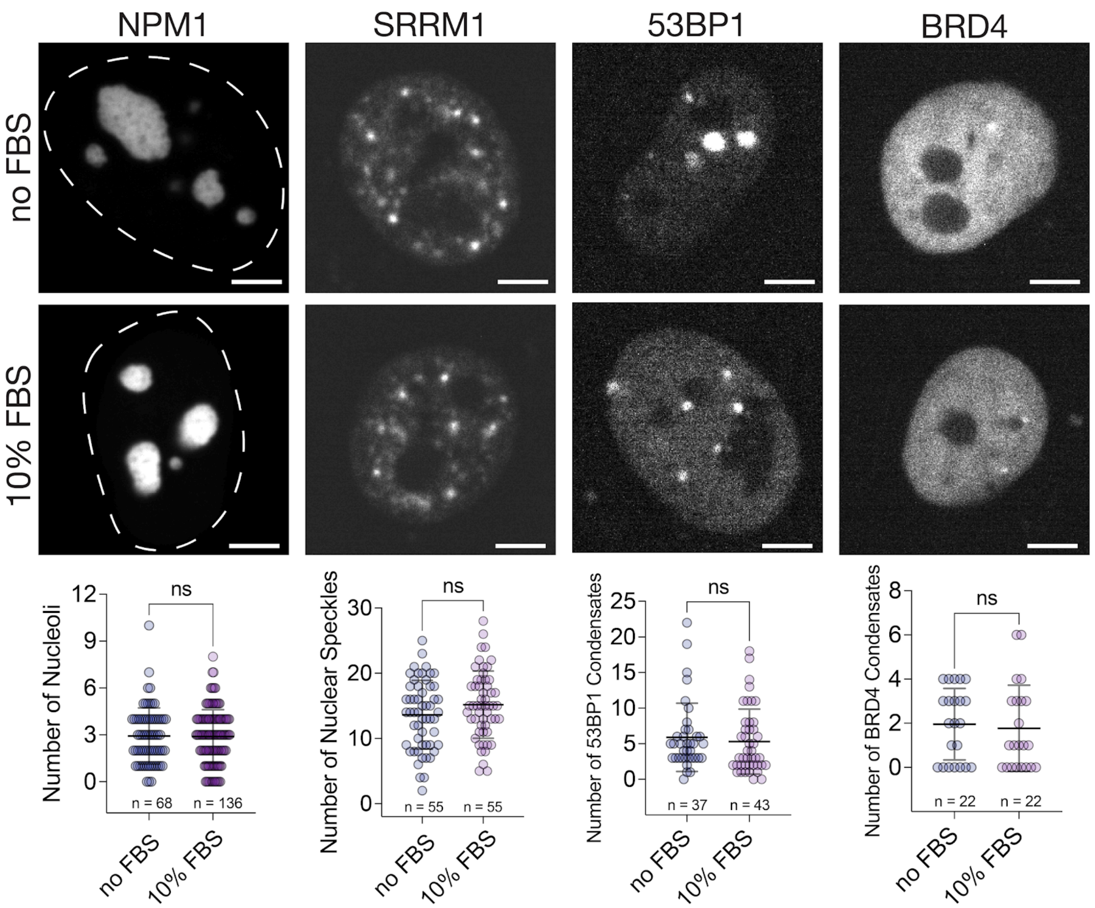

Supplementary Fig. S7. Serum level in media does not affect the number of endogenous condensate tested. Cells were plated 12 hours before imaging in media either containing no FBS or 10% FBS. Error bars are SD with the number of cells indicated in the plots. Statistic significance by t test (two-tailed). Scale bar: 5-μm.

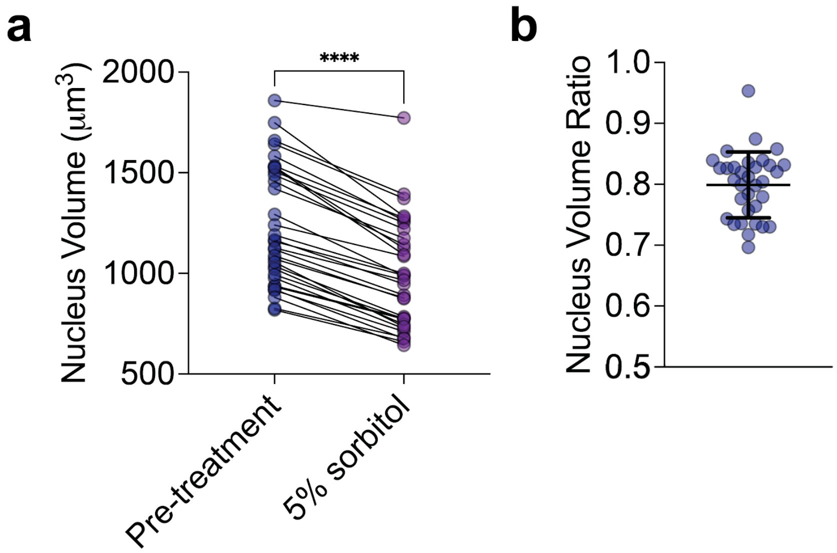

Supplementary Fig. S8. Nucleus volume decrease associated with osmotic compression via 5% sorbitol. On population average, nucleus volume shrinks by 20%, which is used for correcting mGFP-core concentration for phase diagram mapping in Figure 4a. ***$p < 0.001$ by paired t test (two-tailed). Error bar is SD of $n = 33$ cells.

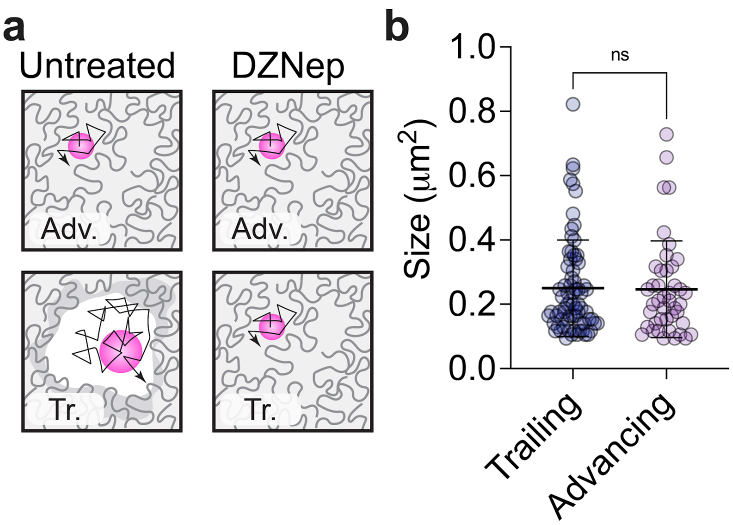

Supplementary Fig. S9. Mechanical characterization of trailing vs advancing half of nucleus upon chromatin decompaction by DZNep. (a) Schematics of chromatin decompaction in cell nucleus undergoing confined migration. (b) Scatterplot shows the size difference of corelet droplets post 3-min activation in trailing vs advancing halves. n.s. $p = 0.9024$ according to unpaired t test (two-tailed). Related to Fig. 3b for untreated cells undergoing confined migration. Error bars are SD of 73 and 43 condensates localized in trailing and advancing halves, respectively.

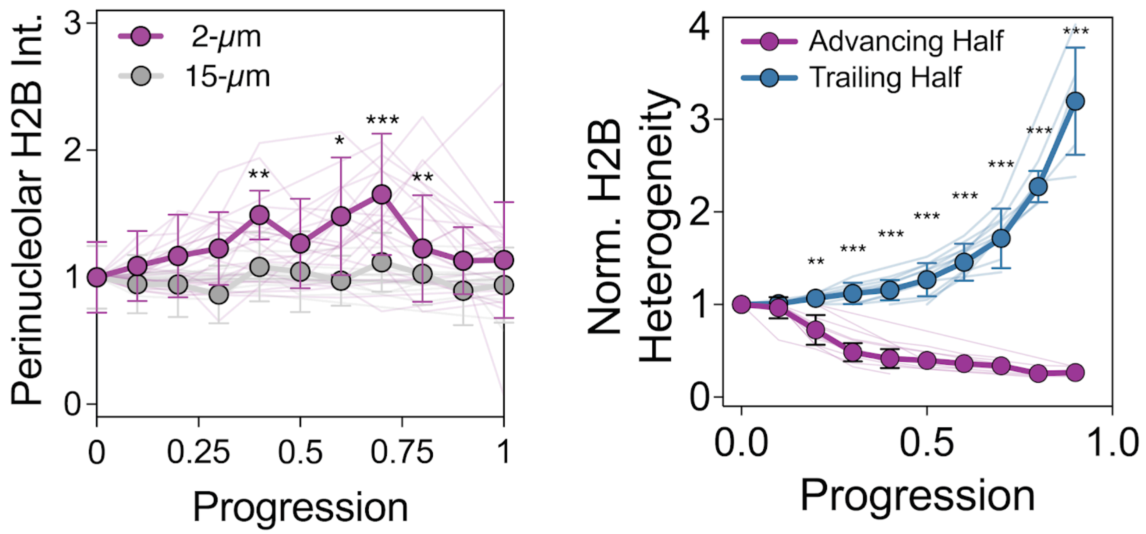

Supplementary Fig. S10. Individual traces of perinucleolar H2B intensity and H2B heterogeneity during migration. Related to Figure 2c-d. (Left) Local compaction of perinucleolar heterochromatin in deformed nuclei undergoing confined migration. Magenta: NPM1-mCherry. Green: H2B-mGFP. Plot shows the average intensity of H2B surrounding the nucleoli over migration progression. n = 23 cells in 2-μm channels and 17 cells in 15-μm channels over N = 3 independent experiments. Error bars are standard deviations, and statistical significance $^{**}p = 0.007$ (at Progression = 0.4), $^{*}p = 0.04$ (at Progression = 0.6), $^{***}p < 0.001$ (at Progression = 0.7) and $^{**}p = 0.006$ (at Progression = 0.8) by one-way ANOVA (two-tailed) comparing 2-μm vs 15-μm across multiple progression time points. Scale bars: 5-μm for upper image, 1-μm for lower cropped images. (Right) Differences in chromatin compaction state in different regions of the nuclei during confined migration. Plot shows the normalized H2B heterogeneity within the trailing (blue curve) and advancing (purple curve) compartment over migration progression. Error bars are standard deviations of 16 cells across N=3 independent experiments. $^{**}p = 0.003$, *** $p < 0.001$ according to one-way ANOVA (two-tailed) comparing advancing half vs trailing half at each progression time point. Scale bar: 5-μm.

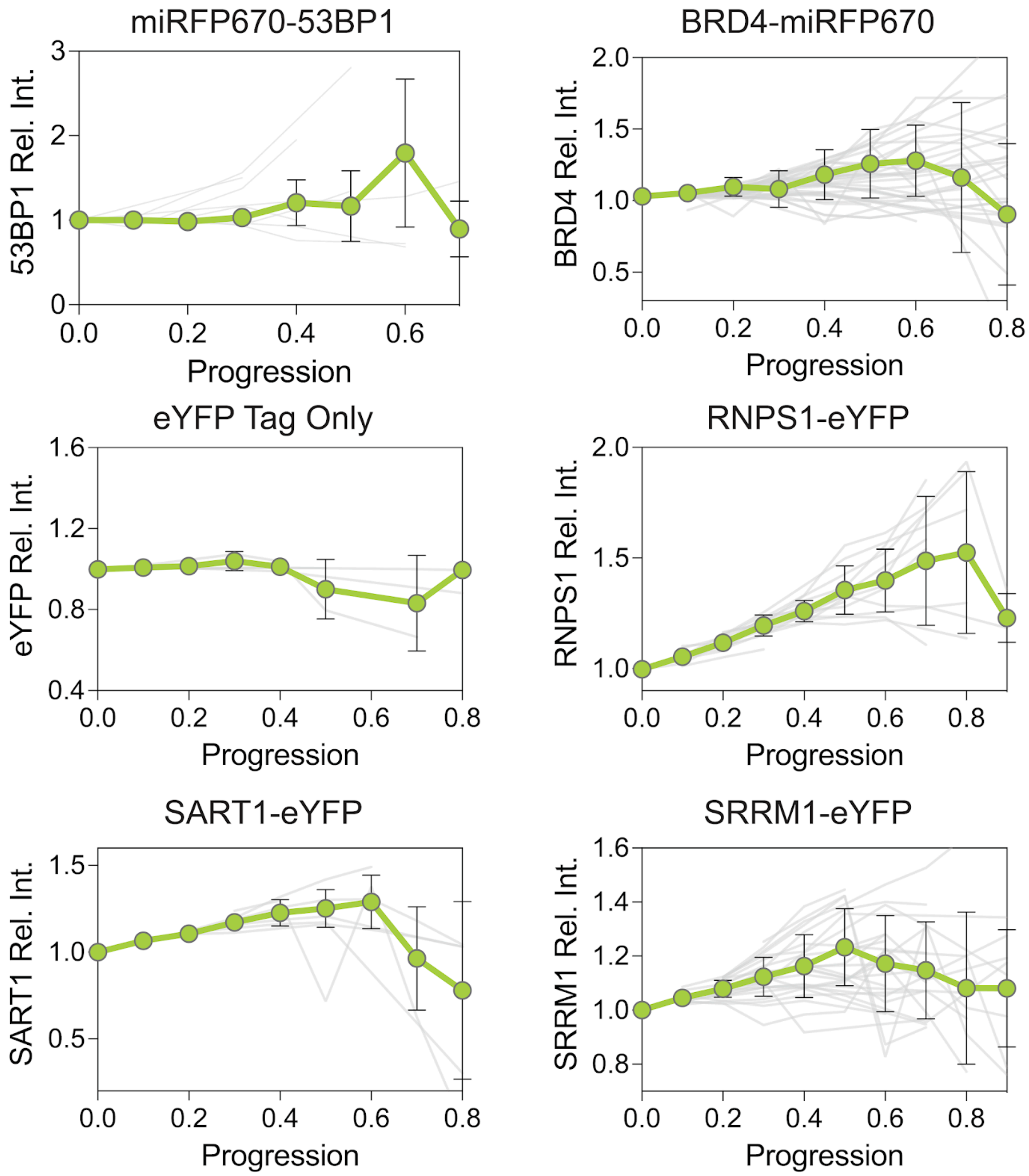

Supplementary Fig. S11. Individual traces of relative intensity in the trailing halves as cells migrate through 2-μm channels. Related to Figure 5. 53BP1 plot: error bars are SD of 14 cells. BRD4 plot: error bars are SD of 33 cells. eYFP tag: n = 4 cells. RNPS1-eYFP, SART1-eYFP and SRRM1-eYFP plots: error bars are SD of 17, 6, 27 cells for RNPS1-eYFP, SART1-eYFP and SRRM1-eYFP, respectively.

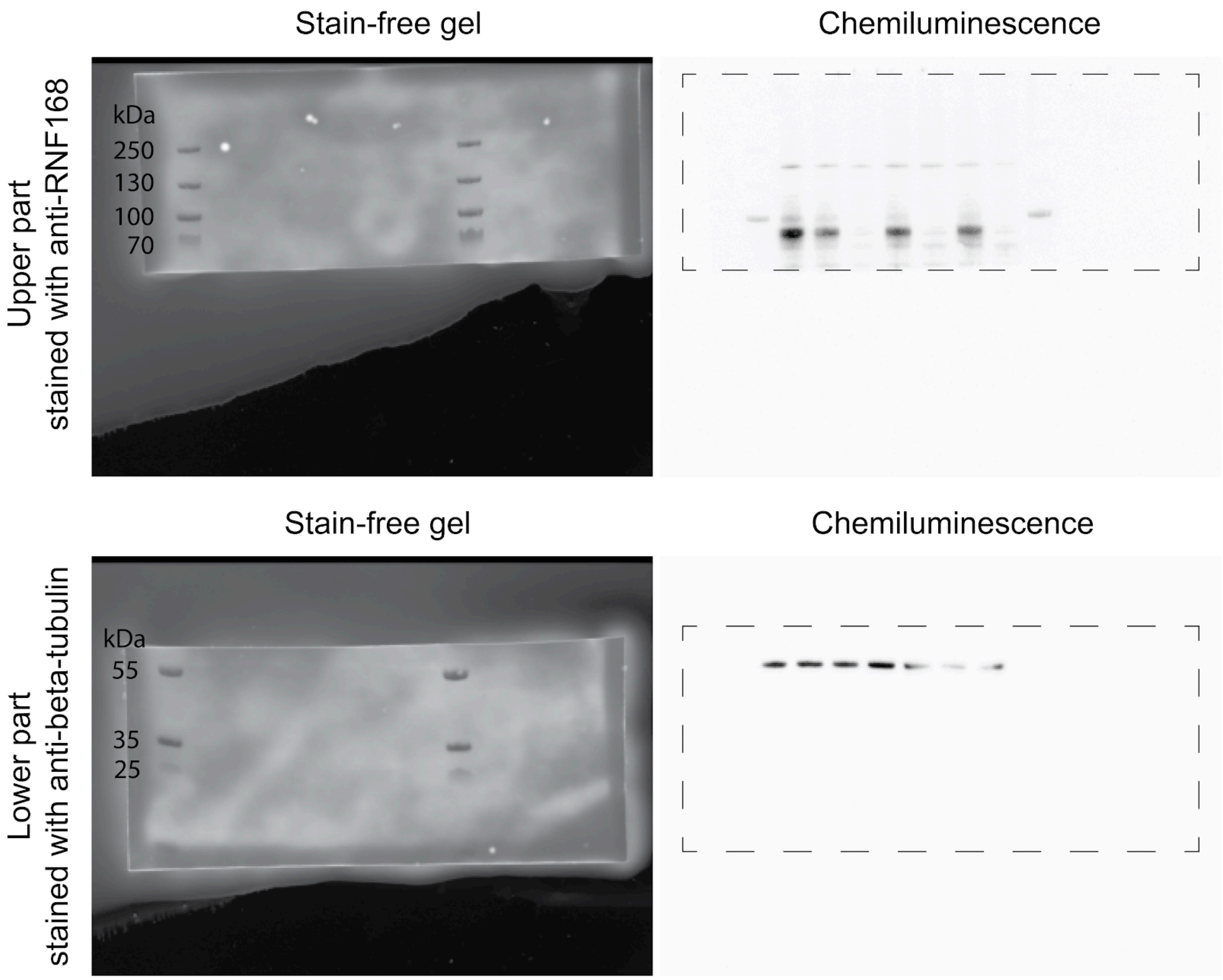

Supplementary Fig. S12. Uncropped western blot. Related to Supplementary Fig. S6a. Upper and lower parts come from the same gel, cut post transfer for separate primary antibody incubation and all subsequent steps.

Supplementary References

1. Shu, T. *et al.* nucGEMs probe the biophysical properties of the nucleoplasm. *bioRxiv* 2021.11.18.469159 (2022) doi:10.1101/2021.11.18.469159.
2. Lee, D. S. W., Wingreen, N. S. & Brangwynne, C. P. Chromatin mechanics dictates subdiffusion and coarsening dynamics of embedded condensates. *Nat. Phys.* **17**, 531–538 (2021).
3. Shimobayashi, S. F., Ronceray, P., Sanders, D. W., Haataja, M. P. & Brangwynne, C. P. Nucleation landscape of biomolecular condensates. *Nature* **599**, 503–506 (2021).
