# Supplementary material for: Chromatin compaction during confined cell migration induces and reshapes nuclear condensates
Source: Nat Commun. 2024 Nov 18;15:9964. doi: 10.1038/s41467-024-54120-5 (PMC11574006; doi:10.1038/s41467-024-54120-5)
Supplement: Supplementary file 2 — Description of Additional Supplementary Information [file 41467_2024_54120_MOESM2_ESM.docx]

**Description of Additional Supplementary Files**

**Supplementary Movie 1**

Nucleolus fusion during confined cell migration. Green: H2B-mGFP. Magenta: NPM1-mCherry.

**Supplementary Movie 2**

*De novo* condensation of light-induced Corelets during cell confined migration. Magenta: FUS_N_-mCherry-sspB, Cyan: Ferritin core.

**Supplementary Movie 3**

De novo condensation of miRFP670-53BP1 during cell confined migration. Green: H2B-mGFP, Magenta: miRFP670-53BP1. Scale-bar: 5-µm.
