# Supplementary material for: Chromatin compaction during confined cell migration induces and reshapes nuclear condensates
Source: Nat Commun. 2024 Nov 18;15:9964. doi: 10.1038/s41467-024-54120-5 (PMC11574006; doi:10.1038/s41467-024-54120-5)
Supplement: Supplementary file 6 — Reporting Summary [file 41467_2024_54120_MOESM6_ESM.pdf]

nature portfolio

Corresponding author(s): Clifford P. Brangwynne

Last updated by author(s): Oct 28, 2024

# Reporting Summary

Nature Portfolio wishes to improve the reproducibility of the work that we publish. This form provides structure for consistency and transparency in reporting. For further information on Nature Portfolio policies, see our Editorial Policies and the Editorial Policy Checklist.

## Statistics

For all statistical analyses, confirm that the following items are present in the figure legend, table legend, main text, or Methods section.

| n/a | Confirmed | |
|---|---|---|
| ☐ | ☒ | The exact sample size ($n$) for each experimental group/condition, given as a discrete number and unit of measurement |
| ☐ | ☒ | A statement on whether measurements were taken from distinct samples or whether the same sample was measured repeatedly |
| ☐ | ☒ | The statistical test(s) used AND whether they are one- or two-sided<br>*Only common tests should be described solely by name; describe more complex techniques in the Methods section.* |
| ☒ | ☐ | A description of all covariates tested |
| ☒ | ☐ | A description of any assumptions or corrections, such as tests of normality and adjustment for multiple comparisons |
| ☐ | ☒ | A full description of the statistical parameters including central tendency (e.g. means) or other basic estimates (e.g. regression coefficient) AND variation (e.g. standard deviation) or associated estimates of uncertainty (e.g. confidence intervals) |
| ☐ | ☒ | For null hypothesis testing, the test statistic (e.g. $F$, $t$, $r$) with confidence intervals, effect sizes, degrees of freedom and $P$ value noted<br>*Give P values as exact values whenever suitable.* |
| ☒ | ☐ | For Bayesian analysis, information on the choice of priors and Markov chain Monte Carlo settings |
| ☒ | ☐ | For hierarchical and complex designs, identification of the appropriate level for tests and full reporting of outcomes |
| ☒ | ☐ | Estimates of effect sizes (e.g. Cohen's $d$, Pearson's $r$), indicating how they were calculated |

*Our web collection on statistics for biologists contains articles on many of the points above.*

## Software and code

Policy information about availability of computer code

| | |
|---|---|
| Data collection | Zeiss Zen Blue software 3.4, Nikon NIS-Elements 5.21 |
| Data analysis | MATLAB_R2023b, GraphPad Prism 10, ImageJ Fiji, and open source software PIVLab, custom code see https://github.com/zzhao97/migration/ |

For manuscripts utilizing custom algorithms or software that are central to the research but not yet described in published literature, software must be made available to editors and reviewers. We strongly encourage code deposition in a community repository (e.g. GitHub). See the Nature Portfolio guidelines for submitting code & software for further information.

## Data

Policy information about availability of data

All manuscripts must include a data availability statement. This statement should provide the following information, where applicable:

- Accession codes, unique identifiers, or web links for publicly available datasets
- A description of any restrictions on data availability
- For clinical datasets or third party data, please ensure that the statement adheres to our policy

All data that support the findings of this study are included in this manuscript and in the Supplementary Information. Source data are provided with this paper.

## Research involving human participants, their data, or biological material

Policy information about studies with human participants or human data. See also policy information about sex, gender (identity/presentation), and sexual orientation and race, ethnicity and racism.

| | |
|---|---|
| Reporting on sex and gender | N/A |
| Reporting on race, ethnicity, or other socially relevant groupings | N/A |
| Population characteristics | N/A |
| Recruitment | N/A |
| Ethics oversight | N/A |

Note that full information on the approval of the study protocol must also be provided in the manuscript.

# Field-specific reporting

Please select the one below that is the best fit for your research. If you are not sure, read the appropriate sections before making your selection.

☒ Life sciences ☐ Behavioural & social sciences ☐ Ecological, evolutionary & environmental sciences

For a reference copy of the document with all sections, see nature.com/documents/nr-reporting-summary-flat.pdf

# Life sciences study design

All studies must disclose on these points even when the disclosure is negative.

| | |
|---|---|
| Sample size | Sample sizes (n) for all other experiments and analyses are defined in the appropriate figure legends. We observe consistent trends or results across samples, providing additional confidence that the sample sizes are sufficient to support the conclusions drawn. |
| Data exclusions | For confined migration experiments, cells were identified for inclusion in various image analyses based on the criteria of complete migration of the cell nuclei through the constriction. |
| Replication | All experiments were performed in independent biological replicates ($N \geq 3$). All attempts at replication were successful. |
| Randomization | Not applicable. The setup for cell line experiments is grouped where different treatments or conditions are directly applied. The experimental design prioritizes replicates and controlled comparisons rather than randomization. |
| Blinding | Not applicable. The experimental readouts are quantitatively assessed across different treatment groups. |

# Reporting for specific materials, systems and methods

We require information from authors about some types of materials, experimental systems and methods used in many studies. Here, indicate whether each material, system or method listed is relevant to your study. If you are not sure if a list item applies to your research, read the appropriate section before selecting a response.

### Materials & experimental systems

| n/a | Involved in the study | |
|---|---|---|
| ☐ | ☒ | Antibodies |
| ☐ | ☒ | Eukaryotic cell lines |
| ☒ | ☐ | Palaeontology and archaeology |
| ☒ | ☐ | Animals and other organisms |
| ☒ | ☐ | Clinical data |
| ☒ | ☐ | Dual use research of concern |
| ☒ | ☐ | Plants |

### Methods

| n/a | Involved in the study | |
|---|---|---|
| ☒ | ☐ | ChIP-seq |
| ☒ | ☐ | Flow cytometry |
| ☒ | ☐ | MRI-based neuroimaging |

## Antibodies

| | |
|---|---|
| Antibodies used | Rabbit anti-53BP1 (#NB-100-305), rabbit (diluted 1:50) anti-β-Tubulin (diluted 1:1000) (Cell Signaling Technology #2146), rabbit anti-RNF168 (Millipore Sigma #ABE367) diluted 1:1000 |
| Validation | In addition to validation provided on the vendors' websties, rabbit anti-53BP1 antibody was validated in immunofluorescence data in |

this manuscript that upon bleomycin treatment, the number of 53BP1 foci stained with anti-53BP1 goes up (Fig. S6b). Rabbit anti-β-Tubulin and anti-RNF168 are validated in western blot data (Fig. S6a) corresponding to the expected size.

## Eukaryotic cell lines

Policy information about cell lines and Sex and Gender in Research

| | |
|---|---|
| Cell line source(s) | The breast adenocarcinoma cell line (female) MDA-MB-231 was a kind gift from Jan Lammerding. Lenti-X 293T cells are commercially available (Takara Bio #632180). |
| Authentication | Lenti-X 293T cells are authenticated by ATCC STR profiling. MDA-MB-231 cell line has not been authenticated. |
| Mycoplasma contamination | Cell lines were not tested for mycoplasma contamination. No contamination was visually observed. |
| Commonly misidentified lines (See ICLAC register) | No commonly misidentified cell lines were used |

## Plants

| | |
|---|---|
| Seed stocks | N/A |
| Novel plant genotypes | N/A |
| Authentication | N/A |
